# Dyke-Davidoff-Masson syndrome: Adult female patient with refractory epilepsy and global cognitive decline

**DOI:** 10.7705/biomedica.7356

**Published:** 2025-05-30

**Authors:** David Ríos, Carlos Cárdenas, Patricia Quintero

**Affiliations:** 1 Unidad de Neurología, Departamento de Medicina Interna, Universidad Nacional de Colombia, Bogotá, D. C., Colombia Universidad Nacional de Colombia Universidad Nacional de Colombia Bogotá, D. C. Colombia; 2 Grupo de Investigación NeuroUnal, Universidad Nacional de Colombia, Bogotá, D. C., Colombia Universidad Nacional de Colombia Universidad Nacional de Colombia Bogotá, D. C. Colombia; 3 Facultad de Medicina, Universidad CES, Medellín, Colombia Universidad CES Universidad CES Medellín Colombia; 4 Facultad de Medicina, Universidad de Antioquia, Medellín, Colombia Universidad de Antioquia Universidad de Antioquia Medellín Colombia; 5 Servicio de Neurología, Hospital Universitario de La Samaritana, Bogotá, D. C., Colombia Hospital Universitario de La Samaritana Hospital Universitario de La Samaritana Bogotá, D. C. Colombia; 6 Servicio de Neurología, Fundación Instituto Neurológico de Colombia, Medellín, Colombia. Fundación Instituto Neurológico de Colombia Fundación Instituto Neurológico de Colombia Medellín Colombia

**Keywords:** Epilepsy, paresis, seizures, epilepsia, paresia, convulsiones

## Abstract

The Dyke-Davidoff-Masson syndrome is characterized by the presence of cerebral hemiatrophy, craneal vault thickening, epileptic seizures, hemiparesis, and cognitive impairment. It is typically diagnosed in childhood and requires specific diagnostic tools for accurate assessment.

This report describes the case of a 19-year-old woman who presented with epileptic seizures and regression in neurodevelopment. She was admitted to the emergency department due to high ictal frequency. During her hospitalization, imaging and electroencephalographic findings were consistent with Dyke-Davidoff-Masson syndrome. Additionally, neuropsychological tests revealed global cognitive impairment. After ten days of hospitalization and five days without epileptic seizures, the patient was discharged. Dyke-Davidoff-Masson syndrome is a rare and often unrecognized condition with high morbidity. Clinicians has the responsibility to identify the key characteristics of the syndrome and perform an adequate differential diagnosis.

## Resumen

El síndrome de Dyke-Davidoff-Masson se caracteriza por hemiatrofia cerebral, engrosamiento de la calota, crisis epilépticas, hemiparesia y discapacidad cognitiva. Usualmente, se diagnostica en la infancia y, para ello, se requieren ayudas diagnósticas específicas.

Se describe el caso de una mujer de 19 años, que se inició con crisis epilépticas y regresión del neurodesarrollo. Ingresó al servicio de urgencias por la gran frecuencia ictal. Durante la hospitalización, se documentaron hallazgos imagenológicos y electroencefalográficos indicativos de síndrome de Dyke-Davidoff-Masson. Además, se practicaron pruebas neuropsicológicas que demostraron discapacidad cognitiva global. Tras diez días de hospitalización y cinco días sin crisis epilépticas, la paciente fue dada de alta.

El síndrome de Dyke-Davidoff-Masson es una entidad rara, poco reconocida y con gran morbilidad. El deber del médico tratante es identificar las características clave del síndrome y llevar a cabo un adecuado diagnóstico diferencial.

The Dyke-Davidoff-Masson syndrome was described by Dyke *et al*. in 1933 and is characterized by cerebral hemiatrophy, calvarial thickening, frontal sinus hyperpneumatization, epileptic seizures, hemiparesis, and cognitive disability ^1^. Although most cases are diagnosed in childhood, close to 20 have been documented in adult patients, mostly females ^2^, with up to 50 years of diagnosis delay ^2^^,^^3^.

Prolonged middle cerebral artery ischemia during gestation (primary syndrome) or in the early postnatal period (acquired syndrome) resulting in cerebral hypermetabolism and reduction of neurotrophic factors have been proposed as the cause of atrophy, leading to clinical manifestations ^4^. Several associated risk factors have been described, including sickle cell anemia, infections, tumors, aortic arch coarctation, trauma, and prolonged febrile crises ^5^. The brain reaches half of its adult size during the first year of life; by the end of the third year, it has acquired three-fourths of its size as it pushes the bone and is partly responsible for the gradual expansion and shape of the adult skull. However, when the brain fails to grow, the bone expands inward, explaining the bone plate abnormalities described in this syndrome ^6^.

Epilepsy usually manifests early, with ictal frequency increasing over time, requiring the use of more than one medication to control epileptic seizures until drug resistance eventually develops. Cognitive impairment varies and can even be associated with neuropsychiatric changes, mostly mood changes with depression and anxiety, and with the use of psychoactive substances ^7^. Hemiparesis is contralateral to hemiatrophy and can be dense. However, it can be overlooked due to preserved function. The least frequent features include short stature and oral and dental changes, such as delayed unilateral dentition, hypoplasia, and taurodontism ^5^.

Diagnosis is primarily determined by clinical and radiological findings. Ideally, brain magnetic resonance imaging should be employed to accurately characterize the typical features.

Subtotal or diffuse cerebral hemiatrophy, paranasal sinus enlargement and hyperpneumatization, and sphenoidal wing, petrous crest and ipsilateral bone hypertrophy are classical findings correlated with hypometabolism in positron emission tomography. However, focal unilateral atrophy of brain peduncles, thalamus, caudate and lenticular nucleus, pons, cerebellum, hypocampus and parahypocampal area can also be found, with prominent cortical sulci and dilated lateral ventricles and cisterns ^8^.

Differential diagnoses to keep in mind include Rasmussen encephalitis, Russell-Silver syndrome, unilateral cerebral polymicrogyria syndrome with hemiatrophy, basal ganglia germinoma, Fishman syndrome, and linear nevus syndrome ^9^.

A therapeutic multidisciplinary approach is required to control seizures using multiple anti-seizure medications. However, the drug regimen of choice remains unclear. Furthermore, some studies report successful outcomes following epilepsy surgery. On the other hand, rehabilitation for motor and cognitive involvement is essential, requiring support from occupational, speech and physical therapy under the guidance of a physiatrist ^10^.

## Case description

A 19-year-old woman living in the rural area of the department of Arauca, Colombia, attended the outpatient service of the *Hospital Universitario de La Samaritana* in Bogotá with a diagnosis of focal epilepsy. During the initial interview, the patient looked disoriented; her companion described multiple epileptic seizures during the previous week. She was transferred to the emergency service, where the medical personnel gave her a dose of benzodiazepine and initiated intravenous antiseizure medication due to suspected non-convulsive status epilepticus.

Upon interviewing the patient and her relative, they reported that her seizures began at the age of three, starting as a febrile seizure (meningoencephalitis was ruled out), followed by a seizure every two months on average. Seizures started with right forearm flexion tonic posturing and gaze supraversion, followed by tonic posturing and clonus of the four limbs, occasionally with loss of sphincter control, no tongue biting, and no recall of the event. Epileptic seizures lasted one to two minutes, leaving the patient drowsy and disoriented for 10 minutes.

She was treated with valproic acid (unknown dose) since she was three years old until she was ten. At that age, she began to receive 250 mg every 8 hours until she was 15, when she started on levetiracetam -1,000 mg every 12 hours- with no ictal frequency improvement. At that time, crises fluctuated between one seizure every two weeks and one seizure every 6 months. A few days before hospitalization, the dose of levetiracetam had been adjusted to 4000 mg/day and she was also prescribed lacosamide 400 mg/day, with worsening of ictal frequency up to even three seizures per day, sometimes with no interictal recovery.

The relative explained that the patient had been assessed by a neurologist only twice in her life, first when she started on antiseizure medications and then two weeks before hospitalization. This situation was due to the limited access to specialized medical services in her region of origin. The patient was a term baby born to non-consanguineal parents through an uncomplicated vaginal delivery.

Neurodevelopmental regression was documented, with milestones achieved at adequate ages but very low academic performance since the start of primary school. Physical examination revealed an acceptable general condition, normal vital signs, childish behavior, dysprosody, bradypsychia, and difficulties in calculation, judgement, and abstraction. No cranial nerve abnormalities were found, and spinal nerve assessment determined left hemiparesis (4+/5), left hyperreflexia (+++/++++), and neutral left plantar response.

### Hospitalization

A contrast brain magnetic resonance angiography, electroencephalogram monitoring, complete blood count, acute phase reactants, and metabolic profile were ordered. The patient had a focal seizure that evolved to bilateral tonic-clonic, consistent with the semiology described above. However, by that time, overt improvement in alertness and attention was already evident.

**Figure 1:**
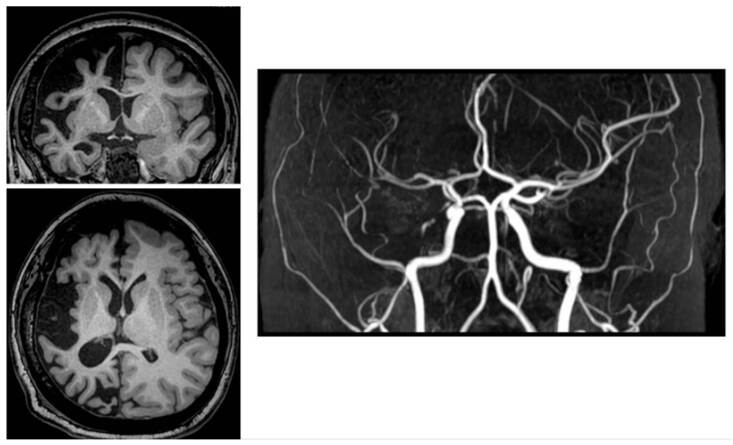
Cerebral magnetic resonance imaging and angiography. Diploe thickening over the right skull convexity, with asymmetric atrophy of the cerebral hemispheres, significantly greater on the right side. It involved all lobes and the midbrain due to hypoplasia of the right cerebral peduncle. Additionally, right hemispheric arteries appear smaller compared to their contralateral counterparts.

Contrast brain magnetic resonance angiography showed evidence of diploe thickening over the right skull convexity atrophy of both brain hemispheres, significantly greater in the right hemisphere. It involved all the lobes and midbrain due to hypoplasia of the right cerebral peduncle, with right hemispheric arteries of smaller caliber than their contralateral counterparts (figure 1).

**Figure 2:**
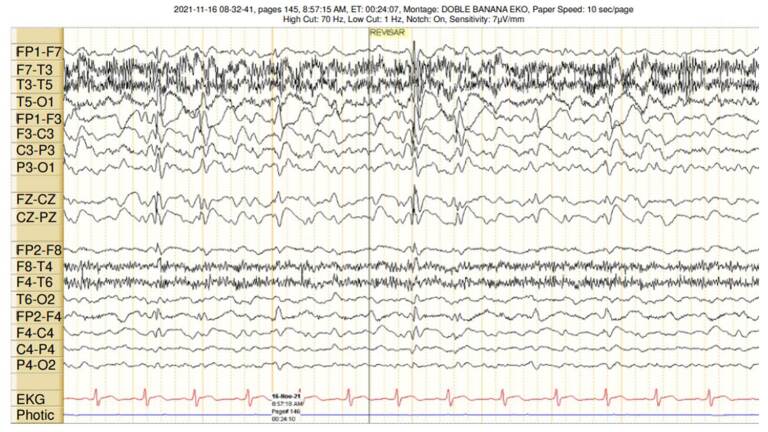
Electroencephalogram monitoring showed a predominantly slowing of the left hemisphere background rhythms and multiple slow wave peak complexes apparently originating in the left hemisphere, with rapid bilateralization (probable false lateralization phenomenon).

Electroencephalogram monitoring showed a predominantly slowing of the left hemisphere background rhythms and multiple slow wave-peak complexes, apparently originating in the left hemisphere, with rapid bilateral propagation. Hemiatrophy clearly predominated on the right side, meaning that the electroencephalographic findings may correspond to a phenomenon of false lateralization (figure 2).

Neuropsychological tests reported impairment in multiple cognitive domains, mainly due to executive function compromise (abstraction, planning, working memory, flexibility), memory and speech challenges (articulation, complex comprehension difficulty, and poor fluency), and impaired social cognition development. Additionally, the Weschler Adult Intelligence Scale (WAIS-IV) showed an extremely low total IQ score of 41 points.

On the first day of hospitalization, the patient initiated a regimen of 500 mg of valproic acid every 8 hours, 2,000 mg of levetiracetam every 12 hours, and 200 mg of lacosamide every 12 hours. Valproic acid was progressively increased to 3,000 mg/day because it was better tolerated and had a greater impact on seizure control. Tapering of the other antiseizure medication and addition of lamotrigine were planned.

After ten days of hospitalization and five days of no epileptic seizures, the patient was discharged with a plan for physiatry-guided rehabilitation, with emphasis on occupational therapy, and follow-up after three months to continue with antiseizure medication adjustments and to define assessment in an epilepsy surgery program.

### Ethical considerations

The patient’s mother authorized the review of her medical history and the publication of this case report by signing the informed consent.

## Discussion

As in the majority of reported cases, our patient demonstrates bone hypertrophy and distinct sulci resulting from a postnatal insult occurring before the age of three ^5^^,^^11^. This case is one of the few reported in which a febrile seizure is considered as the potential etiology of acquired Dyke- Davidoff-Masson syndrome ^12^. In this case, refractory epilepsy could worsen the structural changes and facilitate the onset of global cognitive impairment ^13^. However, in reported cases of concomitant contralateral cerebellar atrophy, potential antegrade transneuronal degeneration has been considered a related cause ^6^.

Although clinical manifestations started in childhood, the diagnosis was delayed due to lack of access to specialized assessment by a neurologist and to the diagnostic tests required to characterize the syndrome. As previously mentioned, there are multiple differential diagnoses and, in this patient, the hemiconvulsion-hemiplegia-epilepsy syndrome is the main differential. Findings that pointed towards a diagnosis of Dyke-Davidoff-Masson syndrome were the initiation of seizures with unilateral tonic posturing and not with hemiclonus, and the fact that they lasted less than two minutes. These findings are not consistent with the definition of the hemiconvulsionhemiplegia-epilepsy syndrome. Moreover, the degree of hemiparesis did not correlate with the degree of cerebral atrophy, something which does happen in hemiconvulsion-hemiplegia-epilepsy. Additionally, calvarial thickening is a hallmark of the Dyke-Davidoff-Masson syndrome. The clinical course was not consistent with the habitual course of the hemiconvulsion-hemiplegia-epilepsy syndrome, in which ictal frequency tends to improve over time, unlike our patient who developed drug-resistant epilepsy ^14^^,^^15^.

As for other differential diagnoses, the presence of skin changes leads to rule out another syndrome associated with cerebral hemiatrophy, namely, Parry-Romberg syndrome. However, the characteristic features of this condition with progressive unilateral hemiatrophy were absent in our patient ^16^.

The Sturge-Weber syndrome is characterized by vascular malformations involving the face, epileptic attacks, glaucoma, mental retardation, and recurrent stroke-like episodes. Examination of our patient did not reveal striking skin findings, recurrent acute neurologic deficit episodes, or characteristic vascular changes on imaging ^17^.

Rasmussen’s encephalitis is a chronic, progressive, immune- mediated disorder not associated with calvarial changes, even though it is characterized by refractory focal epilepsy and cognitive impairment ^18^.

Fishman syndrome is a neurocutaneous disorder that includes unilateral cranial lipoma with ocular lipodermoid lesion. It usually manifests with seizures and could hardly mislead us, given that brain imaging shows hemiatrophy and cortical calcification ^19^. On the other hand, linear nevus syndrome is characterized by facial nevus, recurrent seizures, mental retardation, and unilateral ventricular dilation, which mimics cerebral hemiatrophy but, again, our patient did not present skin or adnexal findings ^20^.

Basal ganglia germinoma includes cystic areas, focal bleeding, and surrounding edema associated with calvarial changes, features that are absent in our case ^21^. Finally, Russell-Silver syndrome involves the classical facial phenotype and normal intelligence, which is not the case in our patient ^22^.

Few cases of Dyke-Davidoff-Masson syndrome have been reported. The treatment mainly consists of physical and occupational therapy, antiseizure medications, and, in cases of drug-resistant epilepsy, hemispherectomy. Emerging palliative therapeutic options include vagus nerve stimulation and responsive neurostimulation of the thalamus, both of which are still awaiting further evidence ^2^^,^^23^.

## Conclusion

Dyke-Davidoff-Masson syndrome is an infrequent condition, practically unknown to most practitioners. It is triggered by early cerebral insult, with heterogenous clinical manifestations. As such, it requires a multidisciplinary approach, advanced imaging modalities, and adequate differential diagnosis. Early recognition is key to improving prognosis.

## References

[1] Dyke C, Davidoff L MC, Masson CB. (1933). Cerebral hemiatrophy homolateral atrophy of the skull and sinuses. Surg Gynecol Obs.

[2] Diestro JDB, Dorotan MKC, Camacho AC, Perez-Gosiengfiao KT, Cabral-Lim LI. (2018). Clinical spectrum of Dyke-Davidoff-Masson syndrome in the adult: An atypical presentation and review of literature. BMJ Case Rep.

[3] Biçici V, Ekiz T, Bingol I, Hatipoglu C. (2014). Dyke-Davidoff-Masson syndrome in adulthood: A 50- year diagnostic delay. Neurology.

[4] Ono K, Komai K, Ikeda T. (2003). Dyke-Davidoff-Masson syndrome manifested by seizure in late childhood: A case report. J Clin Neurosci.

[5] Kalaskar R, Kalaskar AR. (2018). Classical oral manifestations of Dyke-Davidoff-Masson syndrome: A case report with review of the literature. J Korean Assoc Oral Maxillofac Surg.

[6] Güven H, Aytaç E, Comoglu SS. (2014). Dyke-Davidoff-Masson syndrome: A case with additional brain abnormalities. Acta Neurol Belg.

[7] Bhandari SS, Joseph SJ, Sharma IL, Medhi G. (2018). Dyke-Davidoff-Masson syndrome presenting with intellectual disability with behavioral problems and substance use disorder: A case report. Turk Psikiyatri Derg.

[8] Roy U, Panwar A, Mukherjee A, Biswas D. (2016). Adult presentation of Dyke-Davidoff-Masson syndrome: A case report. Case Rep Neurol.

[9] Kuchukhidze G, Unterberger I, Dobesberger J, Embacher N, Walser G, Luef G (2007). Unilateral polymicrogyria with ipsilateral cerebral hemiatrophy: A distinct syndrome?. Epileptic Disord.

[10] Shrestha B. (2013). Acquired cerebral hemiatrophy: Dyke-Davidoff-Masson Syndrome - A case report. Turk Neurosurg.

[11] Solomon GE, Hilal SK, Gold AP, Carter S. (1970). Natural history of acute hemiplegia of childhood. Brain.

[12] Garg RK, Karak B. (1998). Cerebral hemiatrophy: A possible etiological relation with febrile seizures. Indian Pediatr.

[13] Kâlviâinen R, Salmenpera T. (2002). Do recurrent seizures cause neuronal damage? A series of studies with MRI volumetry in adults with partial epilepsy. Prog Brain Res.

[14] Coelho J, Dos Santos TP, Ezequiel M, Luís C, Levy A. (2019). Síndrome de hemiconvulsión- hemiplejía-epilepsia: caso clínico y uso de dextrometorfano. Rev Neurol.

[15] Bhargava H, Dwivedi D. (2020). Hemiconvulsion-hemiplegia-epilepsy syndrome: A case series. J Pediatr Neurosci.

[16] Wong M, Phillips CD, Hagiwara M, Shatzkes DR. (2015). Parry Romberg Syndrome: 7 cases and literature review. AJNR Am J Neuroradiol.

[17] Higueros E, Roe E, Granell E, Baselga E. (2017). Sturge-Weber Syndrome: A review. Actas Dermosifiliogr.

[18] Lagarde S, Boucraut J, Bartolomei F. (2022). Medical treatment of Rasmussen’s encephalitis: A systematic review. Rev Neurol.

[19] Moog U, Dobyns WB. Encephalocraniocutaneous lipomatosis. In: Adam MP, Feldman J, Mirzaa GM, Pagon RA, Wallace SE, Amemiya A. GeneReviews^®^.

[20] Salman S, Fathalla W, Akbari H. (2021). Linear nevus sebaceous syndrome in a child with infantile spasms and focal cortical dysplasia. Cureus.

[21] Vialatte de Pémille C, Bielle F, Mokhtari K, Kerboua E, Alapetite C, Idbaih A. (2016). Basal ganglia germinoma in an adult. World Neurosurg.

[22] Spiteri BS, Stafrace Y, Calleja-Agius J. (2017). Silver-Russell syndrome: A review. Neonatal Netw.

[23] Roa JA, Abramova M, Fields M, Vega-Talbott M, Yoo J, Marcuse L (2022). Responsive neurostimulation of the thalamus for the treatment of refractory epilepsy. Front Hum Neurosci.

